# Occurrence of acute coronary syndrome, pulmonary thromboembolism, and cerebrovascular event in COVID‐19

**DOI:** 10.1002/ccr3.3112

**Published:** 2020-07-22

**Authors:** Yaser Jenab, Nima Rezaei, Behnam Hedayat, Mohammadreza Naderian, Shapour Shirani, Kaveh Hosseini

**Affiliations:** ^1^ Cardiology Department Tehran Heart Center Tehran University of Medical Sciences Tehran Iran; ^2^ Immunology, Asthma and Allergy Research Institute Tehran University of Medical Sciences Tehran Iran; ^3^ Radiology Department Tehran Heart Center Tehran University of Medical Sciences Tehran Iran

**Keywords:** acute coronary syndrome, acute pulmonary thromboembolism, COVID‐19

## Abstract

COVID‐19 causes significant hypoxia along with an exacerbated inflammatory milieu, which may be a trigger for atherosclerotic plaque rupture and/or thromboembolic events. Herein, we presented a case of COVID‐19 presented with acute coronary syndrome followed by pulmonary thromboembolism and cerebrovascular accident.

## Graphical abstract

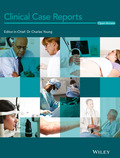

## 1 INTRODUCTION

In December 2019, the coronavirus emerged in the Huanan Seafood Market, Wuhan, China, and rapidly became a worldwide problem.^1^ Now, coronavirus disease (COVID‐19) pandemic is considered to be a real crisis.^2^ About 80% of patients with COVID‐19 seem to have mild or even no symptoms^3^; however, some may experience severe illness.

Arterial and venous thrombotic events have been reported in COVID‐19 patients.^4^ Hypoxia, severe inflammatory response, and baseline traditional risk factors predispose patients to thrombotic events. Cytokine storm in COVID‐19 is the plausible mechanism for thrombotic events. Acute coronary syndrome, pulmonary thromboembolism (PTE), and cerebrovascular accidents (CVA) all may happen in COVID‐19 patients and are probably associated with inflammatory milieu and hemostatic changes.

Here, we report a 70‐year‐old COVID‐19‐positive patient, presenting with acute coronary syndrome, which has been complicated by acute PTE and CVA.

## 2 CASE PRESENTATION

A 70‐year‐old woman was referred to the Emergency Department of Tehran Heart Center with chief complaints of typical chest pain and cold perspiration. Her symptoms had started 4 days earlier, but fever was added to her presentation on the day of admission. Unfortunately, due to the panic among the general population regarding the transmission of COVID‐19, the patient ignored her symptoms at the beginning. She presented just after an increase in the severity and frequency of her chest pain.

Physical examination revealed a body temperature of 38.6°C, a respiratory rate of 20 per minute, a blood pressure of 100/65 mm Hg, a heart rate of 110 bpm, and an O_2_ saturation level of 89%. The patient was ill and appeared toxic; nonetheless, she had a normal consciousness level and was well oriented. Oxygen therapy was initiated. Hopefully, she did not need invasive ventilation. First on‐admission atrial blood gas was as follows: pH: 7.43, PaO_2_ 65 mm Hg, HCO_3_ 19, pCO_2_ 35, and saturation O_2_ 87%.

She has previous history of diabetes mellitus, hypertension, and dyslipidemia. Additionally, she had undergone coronary artery bypass graft surgery 2 years ago in this hospital.

Electrocardiography on admission illustrated sinus tachycardia, generalized ST depressions, and ST elevations in aVR and V1, suggestive for left main or multivessel ischemia. She subsequently underwent echocardiography, which demonstrated a left ventricular ejection fraction (LVEF) of 45%, mild right ventricular (RV) dysfunction, and moderate mitral and tricuspid regurgitation. The patient’s echocardiogram obtained a year before was almost the same, except for mild tricuspid regurgitation.

**Figure 1:**
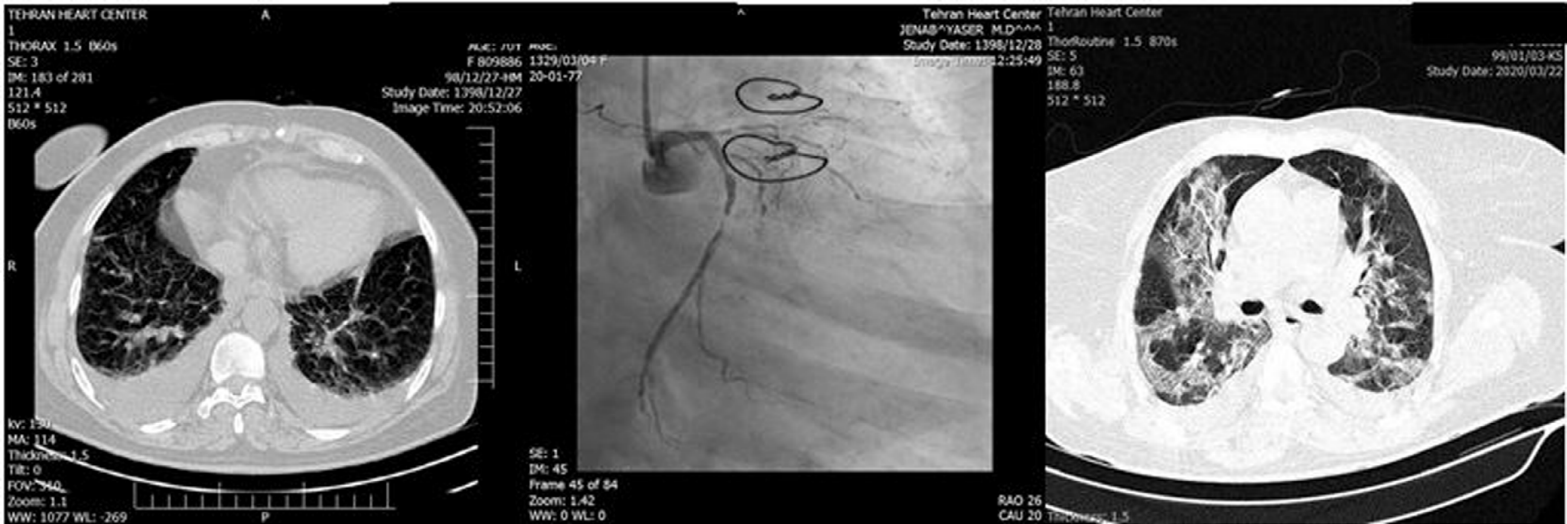
Sorted by time. Left panel: First on‐admission chest computed tomography scan showing bilateral pleural effusion. Middle panel: 10 h after admission, coronary angiogram, post‐CABG, left main is cut, and very significant stenosis in left circumflex artery. Right Panel: Second chest computed tomography scan 3 d later; ground‐glass and patchy areas in favor of COVID‐19 infection

An interventional cardiologist was consulted immediately. Given the patient’s age, fever, and suspicion of COVID‐19, a decision was made to administer full antiplatelet (ASA 300 mg and clopidogrel 300 mg stat doses), anticoagulant (unfractionated heparin; 60 unit/kg stat and 12 unit/kg/hour infusion), and antiischemic therapy and obtain a chest computed tomography (CT) scan. Her chest pain subsided and she became stable; nevertheless, she remained febrile. The patient underwent chest CT scan (Figure 1), which showed mild‐to‐moderate pleural effusion. Based on her symptoms, a real‐time reverse transcription‐polymerase chain reaction (rRT‐PCR) test for COVID‐19 was requested, and hydroxychloroquine was initiated based on infectious disease consultation.

Ten hours later (the next day), the patient experienced an increase in her chest pain. She was transferred to the catheterization laboratory. The anastomosis of the left internal mammary artery to the left anterior descending artery was open, but the anastomosis of the saphenous vein graft to the left circumflex artery (LC_X_) was occluded and the LC_X_ was 90%‐99% stenotic (Figure 1; middle panel). Percutaneous coronary intervention (PCI) was performed on the LC_X_. Postprocedurally, the patient experienced a relief in her chest pain and became stable, but her fever persisted and her O_2_ saturation level was 89%‐90%. Dry coughs became more prominent. The second spiral chest CT scan was compatible with COVID‐19, and the rRT‐PCR result was also positive for COVID‐19. Chest CTs and coronary angiogram sorted by time have been depicted in Figure 1.

Accordingly, lopinavir/ritonavir (KALETRA) was added to her drug regimen, and O_2_ therapy under the supervision of an anesthesiologist was continued. Her laboratory tests revealed a white blood cell count of 10 700, a lymphocyte count of 1070, a C‐reactive protein level of 2.5 (>0.5 mg/dL), a creatinine level of 2.1 mg/dL, a brain natriuretic peptide level of 530 pg/mL, and on‐admission troponin level of 269 ng/L, which increased to 560 ng/L after 2 hours of admission. Her blood culture was negative.

After 10 days of treatment with antiviral drugs, the patient’s symptoms worsened and her O_2_ saturation level dropped to 70%. Her systolic blood pressure dropped to 70‐80 mm Hg, but she was oriented; for some hours, she was on positive inotrope, and hopefully, this was tapered and discontinued afterward. Chest CT scan illustrated diffuse ground‐glass pattern in the parenchyma. This chest CT scan was discernibly worse than the previous one.

**Figure 2:**
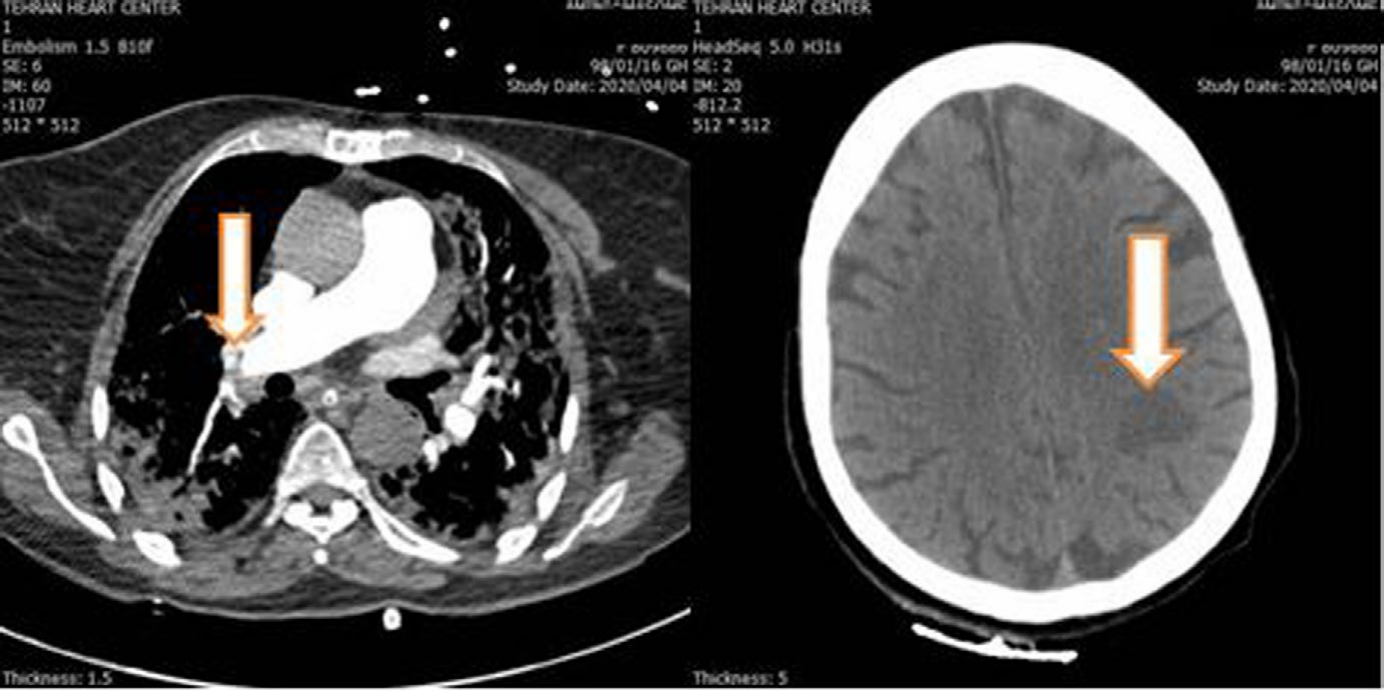
Left panel: Pulmonary thromboembolism in right lobar pulmonary artery, white arrow. Right panel: Brain CT. Probable thromboembolic ischemia in left parieto‐occipital area, white arrow

In the meantime, the patient’s persistent post‐PCI tachycardia, hypoxia, and moderate immobility urged us to investigate other possible causes of hypoxia such as tamponade, mechanical complications of myocardial infarction, and acute PTE. In addition, she experienced weakness and paresthesia of the right arm. Bedside echocardiography showed an LVEF of 40%‐45%, moderate RV dysfunction, moderate mitral regurgitation, and severe tricuspid regurgitation. Pulmonary CT angiography demonstrated bilateral, lobar PTE with an RV/LV of about 0.93 (Figure 2). As the patient had been on prophylactic heparin (5000 unit SC every 8 hours) since PCI, the therapeutic dosage (80 unit/kg stat and 18 unit/kg/hour) was resumed. Unfortunately, in brain CT a hypodense area in left parieto‐occipital area and also semiovale area was noted. It may be an embolic event from thrombotic arterial lesion (because it is unilateral, plaque rupture is probable), and also, it may be due to pressure drop in the territory of a stenotic internal carotid artery that led to watershed infarct. Since the patient was not in good condition, brain MRI was not performed at that time (Figure 2).

Fortunately, she survived and was discharged from the hospital in an acceptable condition and with stable vital signs. The diagnosis of PTE and initiation of therapeutic heparin clearly improved her condition. Stroke rehabilitation was started from hospital and requested in outpatient visits.

## 3 DISCUSSION

The patient was an old woman with multirisk factors suffering from acute coronary syndrome, COVID‐19, PTE, and acute CVA. Recent reports indicated that older patients with cardiovascular disease constitute the highest risk group. This high‐risk group is likely to have coagulopathy; hence, an interim guideline was issued by the International Society on Thrombosis and Hemostasis, suggesting low‐dose prophylactic heparin for the management of coagulopathy.^5^ Venous thromboembolism threatens patients with COVID‐19 and adds the risk of acute respiratory distress syndrome. Venous thromboembolism seems to remain underdiagnosed in COVID‐19 patients. These patients have pulmonary hypertension and RV dysfunction, and small acute PTE may deteriorate the condition.

Pulmonary thromboembolism occurred in 20% of COVID‐19 ICU patients during 1‐month descriptive study by Poissy et al, twice the number in the same duration in 2019. Of note, the proportion of PTE in COVID‐19 was estimated to be twice the number in influenza outbreak.^6^

Large vessel stroke has also been discussed in association with COVID‐19 in another study by Oxley et al^7^ In the recent study by Li et al, a single‐center observational study was performed on 221 COVID‐19 patients. In their registry, 11% developed acute ischemic stroke. Old patients with COVID‐19 were more prone to acute CVA.^8^

Since COVID‐19 patients are prone to thrombotic events, role of empiric therapeutic anticoagulation (intermediate or full dose) for COVID‐19 patients has been proposed but not agreed yet.^4^ Prophylactic anticoagulation was associated with lower mortality in some studies but not others.^9^, ^10^

With respect to our 70‐year‐old female patient infected with COVID‐19, the deterioration in her clinical status with a baseline acute cardiac condition may have been due to a combination of lung injury and cardiac damage. We repeatedly performed echocardiography and detected RV dysfunction and exacerbated tricuspid regurgitation (by comparison with the previous echocardiography findings). Indeed, the patient’s dependence on inotropes made us suspicious of acute PTE. What further obfuscates the picture in patients with COVID‐19 is the presence of tachypnea, tachycardia, hypoxia, and high D‐dimer levels, rendering the differentiation between this infection and PTE challenging. High D‐dimer is associated with a poor prognosis among patients with COVID‐19 whether or not there is concomitant PTE.^10^

We cannot be certain about the exact time of the occurrence of PTE in our patient. It is likely that PTE occurred in the hospital following PCI and her unwanted immobility due to hypoxia and femoral puncture. She was on therapeutic doses of heparin before PCI and prophylactic doses afterward. Another theory may be the coincidence of acute coronary syndrome, COVID‐19, and PTE, which prompted the patient to refer to our emergency department, albeit with a 4‐day delay.

Since COVID‐19 patients are predisposed to thromboembolism, early diagnosis and treatment of such concomitant problems will improve their condition. We should be cautious to ascribe every sign and symptoms during hospitalization to just COVID‐19.

In the time of present case report, no antiviral drug has been proven to be effective in severe coronavirus infection; however, recommendations were in favor of hydroxychloroquine and/or KALETRA depending on patient’s situation. In recent randomized double‐blind study, remdesivir has been associated with reduction in time of clinical improvement.^11^

Alongside coagulopathy, COVID‐19 causes significant hypoxia along with an inflammatory milieu, which may lead to secondary myocardial infarction and also atherosclerotic plaque rupture. Hence, patients with COVID‐19 are predisposed to both embolic and atherosclerotic events, which are associated with baseline inflammation.^12^

## CONFLICT OF INTEREST

The authors have no conflict of interest to declare.

## AUTHOR CONTRIBUTION

MN and BH: gathered all clinical materials and images. YJ: performed coronary angiography and scientific supervision. SS: reported patients' CT scan. NR: revised the manuscript. KH: reviewed the literature and drafted the initial version of manuscript.

## ETHICAL APPROVAL

Ethical Approval Code: IR‐TUMS.VCR.REC.1399.011.
